# Crusted scabies-associated immune reconstitution inflammatory syndrome

**DOI:** 10.1186/1471-2334-12-323

**Published:** 2012-11-26

**Authors:** Mónica Fernández-Sánchez, Marcela Saeb-Lima, Claudia Alvarado-de la Barrera, Gustavo Reyes-Terán

**Affiliations:** 1 Centro de Investigación en Enfermedades Infecciosas, Instituto Nacional de Enfermedades Respiratorias Ismael Cosío Villegas, Calzada de Tlalpan 4502, Colonia Sección XVI, C.P. 14080, Delegación Tlalpan, México D.F

**Keywords:** Crusted scabies, IRIS, HIV, AIDS, ART

## Abstract

**Background:**

Despite the widely accepted association between crusted scabies and human immunodeficiency virus (HIV)-infection, crusted scabies has not been included in the spectrum of infections associated with immune reconstitution inflammatory syndrome in HIV-infected patients initiating antiretroviral therapy.

**Case presentation:**

We report a case of a 28-year-old Mexican individual with late HIV-infection, who had no apparent skin lesions but soon after initiation of antiretroviral therapy, he developed an aggressive form of crusted scabies with rapid progression of lesions. Severe infestation by *Sarcoptes scabiei* was confirmed by microscopic examination of the scale and skin biopsy. Due to the atypical presentation of scabies in a patient responding to antiretroviral therapy, preceded by no apparent skin lesions at initiation of antiretroviral therapy, the episode was interpreted for the first time as “unmasking crusted scabies-associated immune reconstitution inflammatory syndrome”.

**Conclusion:**

This case illustrates that when crusted scabies is observed in HIV-infected patients responding to antiretroviral therapy, it might as well be considered as a possible manifestation of immune reconstitution inflammatory syndrome. Patient context should be considered for adequate diagnosis and treatment of conditions exacerbated by antiretroviral therapy-induced immune reconstitution.

## Background

Crusted scabies is a severe infestation by *Sarcoptes scabiei*. This debilitating form of the disease has been observed mainly in immunosuppressed individuals, while healthy individuals usually present the classic form of scabies [1]. In HIV-infected patients, a wide range of clinical presentations has been described [2], and progression to crusted scabies has been related to CD4 T-cell counts below 150 cells/μL [3].

The positive effects of successful antiretroviral therapy (ART) for the treatment of HIV involve the decrease in HIV-RNA levels and the consequent elevation of CD4 T cell counts. However, as a result of ART-induced immune reconstitution, the proportion of patients who course with inflammatory response and apparent clinical deterioration has been estimated from less than 10% to more than 50% [4], a phenomenon described as Immune Reconstitution Inflammatory Syndrome (IRIS) [5,6]. Even though the association between crusted scabies and HIV has long been reported before the use of ART [7], crusted scabies has not been included among the infections that may be exacerbated by ART-induced immune reconstitution, and thus, it has not been explained as an event of IRIS. Here, we describe an HIV-infected patient initiating ART in whom asymptomatic scabies progressed to crusted scabies in a total period of one week, suggesting a process of crusted scabies-associated IRIS.

## Case presentation

**Figure 1:**
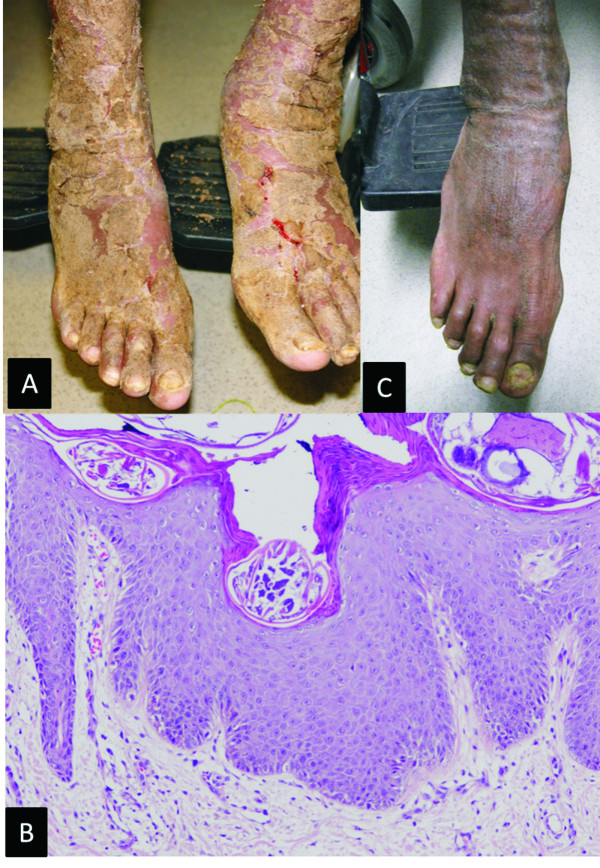
**A) Hyperkeratotic, hyperpigmented crusted plaques with hemorrhagic fissures. B**) Psoriasiform hyperplasia with scarce perivascular superficial lymphocytic inflammatory infiltrate. Numerous fragments of *Sarcoptes scabiei* are present within the stratum corneun. **C**) Resolution of dermatosis after treatment.

A 28-year-old man confined in a crowded prison presented to our institution due to respiratory symptoms. During hospitalization he was subjected to a standardized diagnostic approach including physical examination and a detailed clinical history. Routine laboratory tests, CD4 T cell count and HIV viral load were performed. Diagnosis of late HIV infection (CD4 T-cell count of 164 cells/μL and HIV-RNA of 1 030 082 copies/ml; 6.01 log), disseminated tuberculosis involving lymph node and central nervous system, and cytomegalovirus retinitis were established. Appropriate treatment for these conditions was administrated, and after 13 days in hospital he initiated ART with tenofovir, emtricitabine and efavirenz. His clinical condition improved and 5 days later he was discharged. No skin lesions were visible when he attended a routine visit after 13 days on ART. Interestingly, he presented with disseminated itchy dermatosis characterized by hyperkeratotic, hyperpigmented crusted plaques with hemorrhagic fissures after 20 days on ART (Figure 1A). Microscopic examination of the scale and skin biopsy enabled identification of scabies mites and eggs (Figure 1B). The temporal relation with ART initiation and the rapid progression of infection by *Sarcoptes scabiei*, in presence of an exaggerated inflammatory reaction and atypical inflammatory response in the affected tissues, supported the diagnosis of IRIS [5]. Complete resolution of dermatosis was observed with administration of 3 doses of 12 mg ivermectin given at 1-week intervals, as well as 5-day topical ointment containing balsam of Peru, precipitate sulfur and benzoate butter (Figure 1C). The confinement center received notification of this case, and was also provided with recommendations for the prevention and control of scabies. Days later the patient was discharged home, where he transmitted *Sarcoptes scabiei* to his mother, father and pregnant sister. They all received appropriate treatment leading to remission of the lesions. After three months on ART the patient had a CD4 T-cell count of 281 cells/μl and a viral load of 86 HIV RNA copies/ml (1.93 log). At this point, the diagnosis of IRIS was confirmed by the ART-induced decrease of more than 4 log in the viral load.

## Conclusions

HIV represents a prevalent health problem and scabies is a common parasitic infection of global proportion. Here we present a patient with both infections that coursed with inflammatory response and apparent clinical deterioration soon after antiretroviral therapy initiation, a condition that was interpreted for the first time as crusted scabies-associated immune reconstitution inflammatory syndrome. The atypical presentation of infection by *Sarcoptes scabiei* in a patient responding to ART, preceded by no apparent skin lesions at initiation of ART, suggest that it was an event of “unmasking crusted scabies-associated IRIS”. This condition occurs as a result of unmasking of clinically silent infection, and is characterized by atypical exuberant inflammation and/or an accelerated clinical presentation, indicating a restoration of antigen-specific immunity [8].

Most cases of IRIS related to the use of ART have been associated with bacteria, viruses and fungi. However, the range of infections associated with IRIS is increasing, including now parasitic infections such as leishmaniasis [9], strongyloidiasis [10], schistosomiasis [11] and toxoplasmosis [12]. Here we present evidence that when crusted scabies is observed in the context of immune reconstitution, it might as well be considered among the parasitic infections contributing to episodes of IRIS.

Patients in resource limited settings usually present for HIV clinical care with symptomatic HIV-disease and low CD4 T cell counts. This becomes particularly relevant if we consider that the prevalence of chronic parasitic infections is high in these populations. Scabies is frequent in confinement centers and in hospitals, where outbreaks resulting from exposure to undiagnosed crusted scabies cases have been described [7]. Therefore, consideration of the whole scenario is important for ART initiation in these populations. In addition, an adequate interpretation of conditions exacerbated by ART-induced immune reconstitution is crucial for optimal clinical management of patients.

Despite we are living in the era of ART, we would like to stress that residents of confinement centers and mental hospitals where unhealthy and overcrowded conditions prevail, still do not have access to health care systems. Absence of diagnosis of HIV-infection while our patient was in prison illustrates this notion.

## Consent

Written informed consent was obtained from the patient for publication of this Case report and any accompanying images. A copy of the written consent is available for review by the Series Editor of this journal.

## Abbreviations

HIV: Human immunodeficiency virus; AIDS: Acquired immunodeficiency syndrome; ART: Antiretroviral therapy; IRIS: Immune reconstitution inflammatory syndrome.

## Competing interests

The authors declare that they have no competing interests.

## Authors’ contributions

MFS, MSL and GRT contributed to study conception and design, and participated in acquisition, analysis and interpretation of data. CAB drafted the manuscript, and all authors were involved in the critical review of the manuscript for important intellectual content. All authors have given final approval of the version to be published.

## Pre-publication history

The pre-publication history for this paper can be accessed here:

http://www.biomedcentral.com/1471-2334/12/323/prepub

## References

[B1] Walton SF The immunology of susceptibility and resistance to scabies Parasite Immunol 2010 32 532 540 20626808 10.1111/j.1365-3024.2010.01218.x

[B2] Schlesinger I Oelrich DM Tyring SK Crusted (Norwegian) scabies in patients with AIDS: The range of clinical presentations South Med J 1996 87 352 356 8134858 10.1097/00007611-199403000-00011

[B3] Orkin M Scabies in AIDS Semin Dermatol 1993 12 9 14 8476736

[B4] Müller M Wandel S Colebunders R Attia S Furrer H Egger M Immune reconstitution inflammatory syndrome in patients starting antiretroviral therapy for HIV infection: a systematic review and meta-analysis Lancet Infect Dis 2010 10 251 261 10.1016/S1473-3099(10)70026-8 20334848 PMC4183458

[B5] French MA Price P Stone SF Immune restoration disease after antiretroviral therapy AIDS 2004 18 1615 1627 10.1097/01.aids.0000131375.21070.06 15280772

[B6] Meintjes G Lawn SD Scano F Maartens G French MA Worodria W Elliott JH Murdoch D Wilkinson RJ Seyler C John L van der Loeff MS Reiss P Lynen L Janoff EN Gilks C Colebunders R International Network for the Study of HIV-associated IRIS Tuberculosis-associated immune reconstitution inflammatory syndrome: case definitions for use in resource-limited settings Lancet Infect Dis 2008 8 516 523 10.1016/S1473-3099(08)70184-1 18652998 PMC2804035

[B7] Sirera G Rius F Romeu J Llibre J Ribera M Soriano V Tor J Ferrandiz C Clotet B Hospital outbreak of scabies stemming from two AIDS patients with Norwegian scabies Lancet 1990 335 1227 1971073 10.1016/0140-6736(90)92754-6

[B8] Murdoch DM Venter WD Van Rie A Feldman C Immune reconstitution inflammatory syndrome (IRIS): review of common infectious manifestations and treatment options AIDS Research and Therapy 2007 4 9 http://www.aidsrestherapy.com/content/pdf/1742-6405-4-9.pdf. 10.1186/1742-6405-4-9 17488505 PMC1871602

[B9] Jiménez-Expósito MJ Alonso-Villaverde C Sardà P Masana L Visceral leishmaniasis in HIV-infected patients with non-detectable HIV-1 viral load after highly active antiretroviral therapy AIDS 1999 13 152 153 10207569

[B10] Lanzafame M Faggian F Lattuada E Antolini D Vento S Strongyloidiasis in an HIV-1-infected patient after highly active antiretroviral therapy-induced immune restoration J Infect Dis 2005 191 1027 10.1086/428099 15717283

[B11] de Silva S Walsh J Brown M Symptomatic Schistosoma mansoni infection as an immune restoration phenomenon in a patient receiving antiretroviral therapy Clin Infect Dis 2006 42 303 304 10.1086/499109 16355348

[B12] Tsambiras PE Larkin JA Houston SH Case report. Toxoplasma encephalitis after initiation of HAART AIDS Read 2001 11 615 616 11806173

